# Pre-Injury Adversity, Functional Recovery, and Salivary microRNA Changes After a Dual-Task Exercise in Asians and Pacific Islanders with Mild Traumatic Brain Injury: A Feasibility Study

**DOI:** 10.3390/clinpract16040065

**Published:** 2026-03-25

**Authors:** Hyunhwa Lee, Haehyun Lee, Jinyoung Park, Jessica Gill

**Affiliations:** 1 School of Nursing, University of Nevada, Las Vegas (UNLV), Las Vegas, NV 89154, USA; 2 School of Integrated Health Sciences, University of Nevada, Las Vegas (UNLV), Las Vegas, NV 89154, USA; leeh84@unlv.nevada.edu; 3 School of Nursing, Soonchunhyang University, Cheonan 31151, Republic of Korea; park2025@sch.ac.kr; 4 School of Nursing, Johns Hopkins University, Baltimore, MD 21218, USA; jessicagill@jhu.edu

**Keywords:** mild traumatic brain injury, dual-task exercise, cognitive rehabilitation, psychosocial recovery, salivary microRNA, pre-injury adversity, Asian and Pacific Islanders, health disparities, remote intervention

## Abstract

**Background:** Mild traumatic brain injury (mTBI) is frequently associated with persistent cognitive and psychosocial symptoms, yet biological correlates of recovery remain poorly understood, particularly among Asian and Pacific Islander (API) populations. Pre-injury psychosocial adversity may further shape post-injury recovery trajectories. This pilot study examined associations between participation in a 2-week, home-based, dual-task cognitive–walking intervention (Daily Brain Exercise; DBE) and changes in cognitive, psychological, and salivary microRNA (miRNAs) measures among APIs with and without a self-reported history of mTBI. **Methods:** API participants completed remote cognitive testing (CNS Vital Signs), psychosocial assessments (Neuro-QoL), and saliva collection before and after DBE participation. Salivary RNA was purified, and miRNA expression was profiled using nCounter^®^ Human v3 miRNA Expression Panels (NanoString). Differential expression analyses were conducted using ROSALIND^®^ platform (OnRamp Bioinformatics, San Diego, CA, USA), a cloud-based bioinformatics analysis system, to calculate fold changes and *p*-values. Pre-injury psychosocial adversity was assessed via the Trauma History Screen and examined descriptively as a contextual modifier of functional outcomes. **Results:** Twenty-one APIs (mean age 22.9 years; 76.7% female) were enrolled, including 14 individuals with a self-reported history of mTBI (mean 4.64 years post-injury; 50% with multiple injuries). Following DBE participation, increases in cognitive flexibility and executive function scores were observed in both mTBI and control groups. Additional increases in psychomotor speed, processing speed, sleep disturbance, and depressive symptoms were observed descriptively within the mTBI group. Subgroup analyses suggested variability in pre–post patterns across combinations of mTBI history and pre-injury psychosocial adversity. Exploratory miRNA analyses identified seven miRNAs that were differentially expressed in the mTBI group following DBE (unadjusted *p* < 0.005), including hsa-miR-7-5p, previously reported in association with neurodevelopmental and neurological pathways. **Conclusions:** In this pilot, feasibility-focused study, participation in a brief, home-based, dual-task intervention was associated with descriptive changes in selected cognitive and psychosocial measures among APIs, particularly those with a history of mTBI and pre-injury adversity. The observed subgroup patterns warrant confirmation in adequately powered, controlled studies. Exploratory changes in salivary miRNAs co-occurred with functional improvements, thus generating a hypothesis for a future investigation.

## 1. Introduction

Mild traumatic brain injury (mTBI), commonly referred to as concussion [1], accounts for approximately 80% of all traumatic brain injuries (TBIs) [2]. TBI broadly refers to closed head trauma resulting in temporary or permanent neurobiological impairments and affects millions of individuals. In the United States alone, there are an estimated 4.8 million cases annually, contributing to a global incidence of approximately 69 million [3]. The economic impact is equally staggering; the estimated lifetime economic burden in the United States is projected to be over $750 billion [3].

mTBI is conventionally diagnosed based on the three clinical criteria: (1) loss of consciousness shorter than 30 min; (2) post-traumatic amnesia shorter than 24 h; and (3) injury scores from 13 to 15 on the Glasgow Coma Scale (GCS) [4]. Although most individuals recover within weeks to months, more than 40% experience persistent cognitive, psychosocial, and sensorimotor symptoms that may interfere with daily functioning. These ongoing difficulties can include impairments in attention, executive function, processing speed, sleep, mood regulation, and physical balance [5,6,7], contributing considerable individual and societal burden.

Recovery following mTBI is highly heterogeneous and influenced by multiple factors beyond injury characteristics alone. While traditional prognostic indicators such as injury severity and number of injuries are commonly considered, emerging evidence suggests that pre-injury health and contextual factors may better capture long-term post-injury recovery trajectories. In particular, psychosocial and environmental factors may influence symptom persistence and functional outcomes, even years after the initial injury.

Pre-injury psychosocial adversity, including exposure to trauma, chronic stress, or early life adversity (ELA) [8], has been increasingly recognized to be associated with vulnerability to prolonged impairment post-injury. Consistent with the Stress–Diathesis Model [9], such adversity may confer vulnerability to greater symptom burden or slower recovery following neurological insult, without necessarily reflecting differences in injury severity. However, empirical research examining the role of pre-injury adversity in mTBI recovery remains limited [7,10].

Racial and ethnic minority populations are disproportionately affected by systematic barriers to healthcare access, stigma, and underutilization of rehabilitation services following an mTBI [11,12,13]. Disparities further compound the complexity of mTBI outcomes. Compared with non-Hispanic White populations, racial and ethnic minority groups with brain injuries frequently experience significantly poorer long-term functional outcomes. These include persistent impairments in activities of daily living (ADLs), reduced engagement in leisure activities, and markedly lower rates of return to work or school [14]. The Asian and Pacific Islander (API) population represents one of the fastest-growing yet most understudied racial groups in the United States [15]. Existing data suggest that APIs may experience challenges related to delayed diagnosis, underreporting of symptoms, and limited access to post-acute care [13,14,16,17]. Despite these documented disparities, APIs remain underrepresented in mTBI rehabilitation research, limiting the generalizability of current evidence and the development of culturally responsive interventions. The inclusion of APIs in mTBI research is therefore essential to improve representation and inform equitable care delivery [17,18].

Rehabilitation strategies targeting both cognitive and physical domains have shown promise in neurological population [19]. Dual-task cognitive-physical training, which integrates simultaneous cognitive and motor demands [19], has been shown to engage divided attention and cortico-striatal circuits [20] and associated with in attention, executive function, and motor coordination in populations with neurological and age-related impairments. Emerging studies suggest that similar approaches may be feasible for individuals with an mTBI; however, most existing interventions are resource-intensive, delivered in person, and rarely consider psychosocial context or biological correlates of recovery [20,21].

Advances in peripheral biomarker research, including salivary microRNAs (miRNAs), offer an opportunity to explore biological signals associated with functional change. miRNAs are small, non-coding RNA molecules involved in gene regulation and have been reported in association with neurodevelopmental and neurological processes (e.g., Wang et al., 2012 [10]). While salivary miRNAs have been proposed as accessible peripheral indicators, their role in mTBI recovery remains exploratory and incompletely understood.

**Figure 1:**
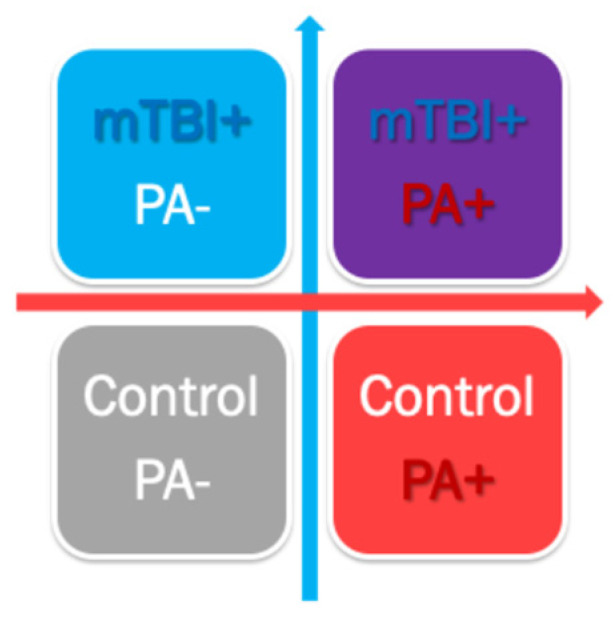
Four subgroups according to mTBI and PA statuses.

The purpose of this study was to examine associations between participation in a 2-week, home-based, dual-task cognitive-walking program—Daily Brain Exercise (DBE)—and changes in cognitive, psychosocial, and exploratory salivary miRNAs measures among APIs with and without a self-reported history of mTBI. Pre-injury psychosocial adversity was examined as a contextual modifier of functional outcomes (Figure 1). Specifically, we aimed to (1) evaluate the feasibility and preliminary efficacy of DBE in APIs; (2) examine patterns of cognitive and psychosocial functional changes in the mTBI group relative to healthy controls; (3) explore salivary miRNAs in association with functional clinical change; and (4) assess the modifying role of pre-injury psychosocial adversity on functional outcomes. This study was designed as a feasibility and hypothesis-generating investigation rather than a definitive test of intervention efficacy, with the goal of informing future controlled and scalable rehabilitation research.

Because most mTBI cases do not result in emergency department admission, neuroimaging abnormalities, or prolonged clinical monitoring, reliance solely on hospital-based cohorts may underestimate longer-term variability in functional outcomes. Community-dwelling individuals with remote mTBI histories therefore represent an important yet under-characterized population in rehabilitation research. Particularly among young adults, early symptom improvement may reduce recognition of persistent or subtle difficulties. Nevertheless, accumulating literature suggests that a subset of individuals with so-called “uncomplicated” or imaging-negative mTBI report relatively poorer cognitive, sleep, or psychosocial outcomes years after injury compared with non-injured peers. Accordingly, the present study adopted a community-based recruitment strategy to capture the functional variability that may not be routinely represented in clinic-based cohorts.

## 2. Conceptual Frameworks

This study was guided by the National Institutes of Health Symptom Science Model (NIH-SSM) [22], which conceptualizes symptoms as complex, observable phenotypes emerging from dynamic interactions between biological, clinical, and psychosocial factors. Within this framework, cognitive and psychosocial symptoms following an mTBI are viewed as measurable outcomes that may be influenced by both the primary injury-related factors and pre-existing contextual vulnerabilities.

Pre-injury psychosocial adversity was incorporated as a contextual factor within NIH-SSM to capture life-course influences that may shape symptom expression and recovery experiences after an mTBI. This approach is further informed by the Stress–Diathesis Model [9], which posits that individuals with greater biological or psychosocial “diathesis” (e.g., exposure to early life adversity or chronic stress) may experience heightened symptom burden or altered recovery trajectories following neurological stressors, such as an mTBI, without implying differences in injury severity or underlying neuropathology.

We also draw upon principles of experience-dependent neuroplasticity [23,24] that emphasize the brain’s capacity for functional reorganization in response to targeted, repetitive activity. Dual-task training integrates concurrent cognitive and motor demands and has been associated with improvements in attention, executive function, and motor coordination in neurological and aging populations [19,21,25]. In the context of mTBI, such approaches may offer a feasible means of targeting functions domains commonly reported as impaired, particularly when delivered in an accessible, home-based format.

Finally, salivary miRNAs were included as exploratory peripheral indicators of biological processes potentially associated with functional change. miRNAs are non-coding RNA molecules involved in post-transcriptional gene regulation and have been reported in association with neurodevelopmental and neurological processes in the prior literature. In the present study, miRNAs were examined descriptively to identify candidate signals for future validation.

Collectively, these frameworks provide a conceptual foundation for examining functional recovery following an mTBI while acknowledging the influence of psychosocial context and the exploratory nature of peripheral biomarker assessment in the present study.

## 3. Materials and Methods

### 3.1. Study Design

This pilot study employed a pre-test–post-test design to examine changes in cognitive, psychosocial, and exploratory biological measures associated with participation in the DBE—a 2-week, home-based, dual-task intervention. API participants with a self-reported history of mTBI were compared descriptively with age- and gender-matched API participants without a history of mTBI. Assessments were remotely conducted at baseline (pre-intervention) and immediately following the 14-day intervention period (post-intervention). This pilot study was intended to evaluate feasibility and characterize observed patterns of change before and after the DBE participation. Participant recruitment and data collection were conducted between January 2021 and June 2021.

### 3.2. Participants and Recruitment

The study was conducted in accordance with the Declaration of Helsinki and was approved by the Institutional Review Board at the University of Nevada, Las Vegas (UNLV) (Protocol No. 1405771). All participants provided informed electronic consent prior to enrollment. Participants were recruited through digital and paper-based flyers, social media postings, and word-of-mouth. Inclusion criteria for the mTBI group were the following: (1) adults aged 18–35 years, (2) a self-reported history of mTBI based on screening with the Ohio State University TBI Identification Method (OSU TBI-ID) [26], and (3) the ability to complete remote assessments and home-based exercises. Exclusion criteria included a history of or active major neurological or neuropsychiatric conditions unrelated to an mTBI. API participants without a history of mTBI were recruited as a comparison group and matched to the mTBI group by age and gender to reduce demographic confounding in outcomes.

Following eligibility screening conducted via videoconferencing (Zoom), participants were offered one of two participation pathways: (a) a one-time remote functional assessment (*n* = 3), or (b) participation in the full 2-week DBE program with pre- and post-test remote assessments (*n* = 18). Participants completing the one-time remote assessment received USD 40 in compensation. Intervention participants received USD 40 following the baseline remote assessment and an additional USD 60 upon completion of the 2-week DBE protocol and post-intervention remote assessment. Each remote assessment session required approximately 60 min to complete.

### 3.3. Pre-Injury Psychosocial Adversity

Pre-injury psychosocial adversity was assessed using the Trauma History Screen (THS), a 13-item self-report instrument capturing lifetime exposure to high-magnitude stressors (HMSs), including accidents, natural disasters, physical or sexual abuse, or other traumatic events [27]. For each endorsed event, respondents indicated frequency and associated distress. Indices derived from the THS included (a) the total number of traumatic stressors (TS) and (b) the presence of persistent post-traumatic distress (PPD), which are consistent with criteria of post-traumatic stress disorder (PTSD) by the Diagnostic and Statistical Manual of Mental Disorders (DSM). The TS and PDD variables were used to stratify participants into subgroups for exploratory analyses. Participants were divided into the adversity-positive (PA+) or adversity-negative (PA−) group based on a median split of the TS and PPD scores.

### 3.4. Intervention: Daily Brain Exercise (DBE)

The DBE protocol was adapted from the dual-task training developed by Silsupadol et al. (2009) [25]. The intervention consisted of 14 consecutive days of 15-min dual-task sessions. Each session required the simultaneous execution of a 15-min motor task and three sequential 5-min cognitive tasks:

• Motor tasks: Tandem walking or figure-eight walking performed in a safe indoor or outdoor environment
• Cognitive tasks: Mental arithmetic (e.g., serial subtraction), verbal fluency, and memory recall exercises.

**Figure 2:**
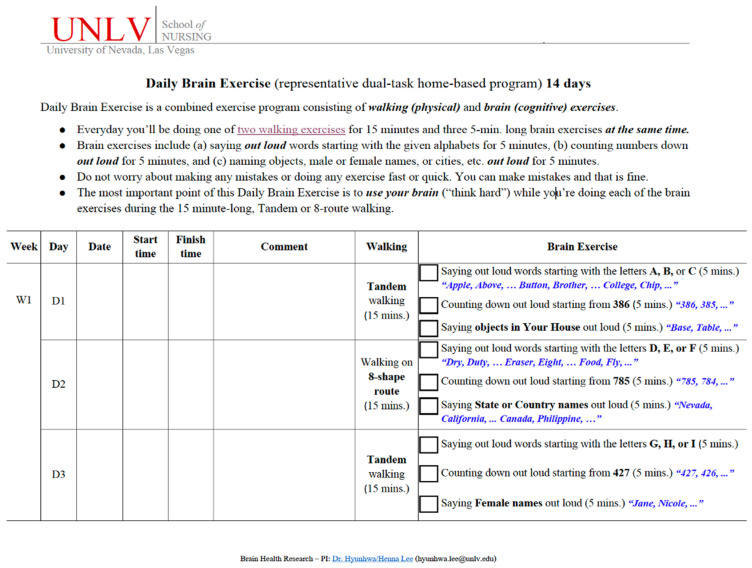
Fourteen-day Daily Brain Exercise Program Log: Each subject received a printed copy and an electronic copy of the following low (its first page is shown below) that guided their 14-day dual-task cognitive–physical exercise.

Intervention training was delivered remotely via videoconferencing with a standardized education session, supplemented by a prerecorded instruction video (link or https://youtu.be/BXLGDwAB8uI, accessed on 17 March 2026). Participants received daily reminders via text message or email and maintained an exercise log throughout the intervention period (Figure 2).

### 3.5. Measures

The following self-report measures were administered via secure Qualtrics online survey links.

#### 3.5.1. mTBI-Related Symptoms

mTBI-related symptoms were assessed using two validated self-report instruments: the Rivermead Post-Concussion Symptom Questionnaire (RPQ) [28] and the Neurobehavioral Symptom Inventory (NSI) [29]. The RPQ is a 16-item measure that assesses post-concussion symptom severity, primarily oriented toward somatic and cognitive symptom burdens. The RPQ demonstrates high test–retest reliability (*r* = 0.87 to 0.91) and is often used for symptom change over time following an mTBI. The NSI is a 22-item instrument with a broader scope, assessing neurobehavioral symptoms commonly associated with an mTBI across physical, cognitive, affective, and sensory domains. The NSI demonstrates excellent internal consistency (*r* = 0.77 to 0.93).

#### 3.5.2. Cognitive Function

Cognitive performance was assessed using CNS Vital Signs (CNS-VS) Remote Testing, a computerized neuropsychological battery administered remotely. CNS-VS evaluates multiple cognitive domains, including memory, attention, psychomotor speed, reaction time, and cognitive flexibility [30]. CNS-VS has demonstrated good reliability and validity across diverse clinical and community populations and is suited for repeated assessments.

#### 3.5.3. Psychosocial Function

Psychosocial functioning was measured using selected domains from the Quality of Life in Neurological Disorders (Neuro-QoL) measurement system [31], including depression, anxiety, sleep disturbance, fatigue, positive affect and well-being, and social participation. Neuro-QoL measures are designed to provide efficient and responsive indicators of health-related quality of life in neurological populations.

### 3.6. Salivary microRNA Analysis

Saliva samples were collected using Oragene RE-100 kits (DNA Genotek, Ottawa, ON, USA) mailed to participants with written instructions and prepaid return packaging. Participants were instructed to refrain from eating, drinking, smoking, chewing gum, or brushing their teeth for at least one hour prior to sample collection. Samples were returned by mail and stored at −70 °C until processing in the UNLV Applied Biobehavioral Research Laboratory following heat incubation for stabilization.

Salivary RNA was purified using the Norgen Saliva/Swab RNA Purification Kit (Norgen Biotek Corp., Thorold, ON, Canada). RNA concentration and quality were assessed using the NanoDrop™ One/OneC Microvolume UV–Vis Spectrophotometer. miRNA expression profiling was performed using nCounter^®^ Human v3 miRNA Expression Panels (NanoString Technologies, Seattle, WA, USA). Differential expression analyses were performed using ROSALIND^®^ software, which normalized the expression data and generated fold change estimates and *p*-values.

### 3.7. Statistical Analysis

Statistical analyses were conducted using SPSS (version 28.0). Descriptive statistics were used to summarize participant characteristics and outcome measures. Between-group comparisons (mTBI vs. control; PA-based subgroups) were conducted using independent-samples *t*-tests and one-way analysis of variance (ANOVA) with Bonferroni-adjusted post hoc tests. Within-group pre–post intervention changes were examined using paired-samples *t*-tests. Statistical significance was set at *p* < 0.05.

Salivary miRNA analyses were exploratory in nature and employed unadjusted *p*-values, consistent with discovery-oriented pilot studies.

## 4. Results

The results are presented descriptively to characterize observed patterns within and between groups.

### 4.1. Sample Characteristics

A total of 21 Asian and Pacific Islanders (APIs) participated in the study (*N* = 21; Table 1). The mean age of the sample was 22.9 years, and two-thirds of participants were female (66.7%). Participants represented diverse API backgrounds, including Filipino (28.6%), Korean (14.3%), and mixed Asian ethnicity (33.3%).

Approximately two-thirds of participants (66.7%) self-reported a history of mild traumatic brain injury (mTBI), with an average of 4.64 years since the most recent injury; half of the mTBI group reported multiple injuries (50%). The most common mechanisms of injury were sports-related activities (57.1%), followed by motor vehicle accidents and daily-life injuries.

No statistically significant differences were observed between participants with and without a history of mTBI with respect to age, sex, education level, employment status, marital status, or income. With respect to mTBI-related symptoms, RPQ total scores were significantly higher in the mTBI group compared with controls, whereas NSI scores did not statistically differ between groups.

For pre-injury psychosocial adversity, the current API sample reported the mean HMS score of 9.2, exceeding levels previously reported in general young adult populations (6.1) [27]. When comparing groups, only the derived THS-PPD score was significantly higher in the mTBI group compared with the controls. Based on median splits of TS and PPD scores, participants were categorized into four subgroups (Figure 1): (a) pre-injury adversity-positive with mTBI (PA + mTBI; *n* = 9), (b) pre-injury adversity-negative with mTBI (PA − mTBI; *n* = 5), (c) pre-injury adversity-positive controls (PA + control; *n* = 2), and (d) pre-injury adversity-negative controls (PA − control; *n* = 5).

### 4.2. Baseline Cognitive and Psychosocial Functioning Before the DBE

Baseline cognitive and psychosocial functioning differed significantly across the four subgroups defined by mTBI history and pre-injury adversity (Supplementary Table S1). PA + mTBI subgroup exhibited a higher self-reported burden across multiple psychosocial domains at baseline, including cognitive difficulty, sleep disturbance (*p*’s < 0.01), cognitive dysfunction, anxiety, depression, emotional and behavioral dyscontrol, and fatigue (*p*’s < 0.05), compared with the other subgroups. This group also reported the lowest levels of participation in social roles and activities (*p* < 0.05).

Post hoc analyses with Bonferroni correction indicated significantly worse anxiety in the PA + mTBI group and more sleep disturbance in the PA + mTBI and PA + control groups, all compared with the PA − control group. Positive affect and well-being were significantly lower in PA + control compared with the PA − mTBI group per post hoc analysis only.

No statistically significant differences across subgroups were observed at baseline for objective cognitive performance as measured by CNS-VS, indicating that baseline cognitive test performance was comparable despite marked differences in self-reported psychosocial functioning. No differences were also observed for mTBI-related symptom scores (RPQ or NSI).

### 4.3. Between-Group Differences After the Daily Brain Exercise

Following completion of the 2-week DBE program, exploratory between-group comparisons were conducted (Supplementary Table S2). The PA − mTBI subgroup demonstrated higher psychomotor speed scores on the CNS-VS (*p* < 0.05), especially compared with the PA + mTBI subgroup, based on post hoc analyses. The PA − mTBI group reported significantly lower Neuro-QoL cognitive dysfunction scores compared with the PA − control group (*p* < 0.05).

No other statistically significant between-group differences were observed across cognitive, psychosocial, or symptom-based measures following the DBE program. Significant improvements in depression and sleep disturbance were observed only in the mTBI group. Improvements in psychosocial functioning were the most pronounced among participants with both an mTBI and pre-injury adversity, indicating differential responsiveness to the intervention.

### 4.4. Within-Group Changes in Cognitive and Psychosocial Functioning After the Daily Brain Exercise

**Figure 3:**
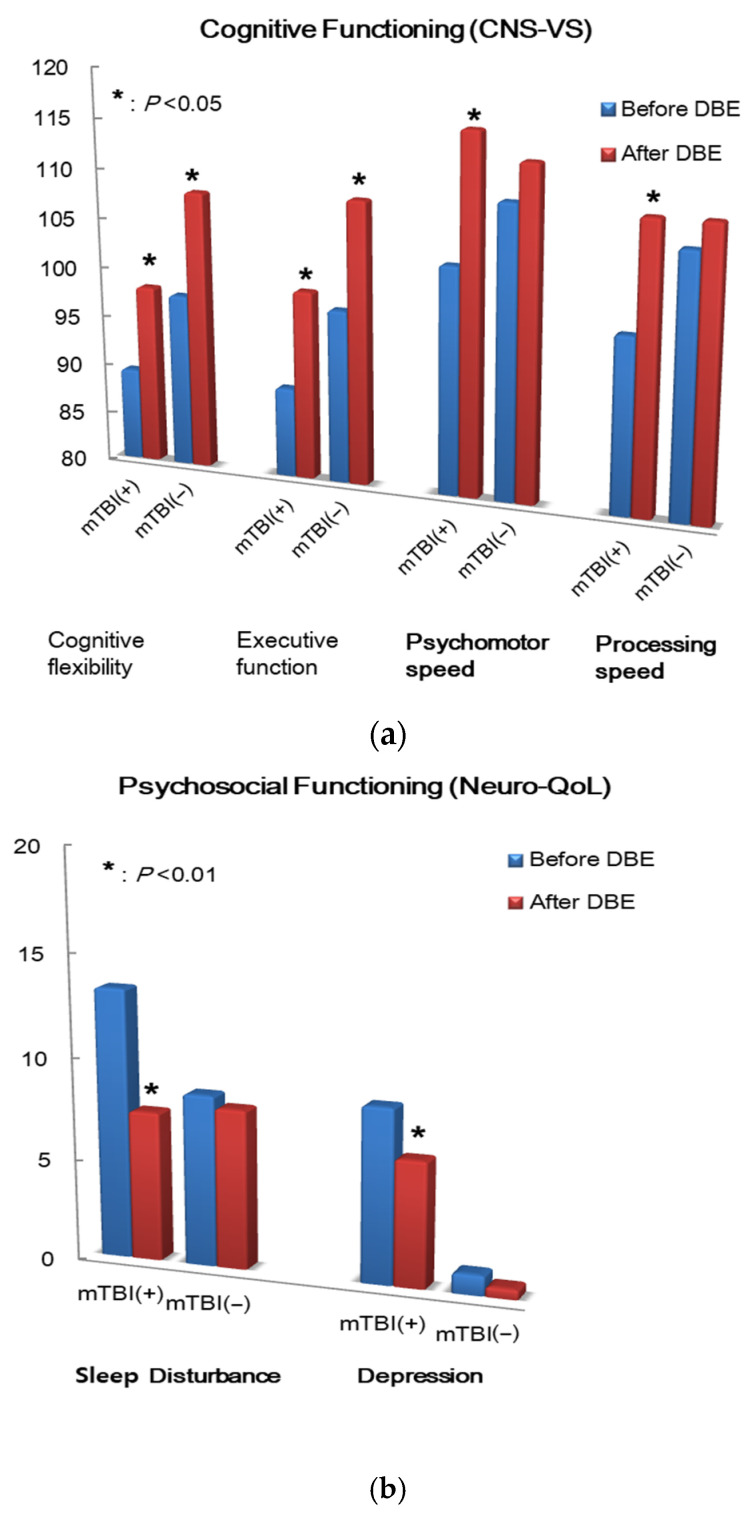
Significant functional changes in mTBI and control groups before and after the DBE. (**a**) Cognitive functioning changes. (**b**) Psychosocial functioning changes.

Within-group pre-post comparisons were conducted to characterize patterns of change following the DBE participation (Figure 3). While both the mTBI and control groups showed improvements in objectively measured cognitive functions, i.e., cognitive flexibility and executive function, only the mTBI participants had additional gains in psychomotor and processing speed (*p*’s < 0.05), as well as improvements in perceived depression, sleep disturbance, and social roles (*p*’s < 0.01).

#### 4.4.1. PA + mTBI Subgroup

**Figure 4:**
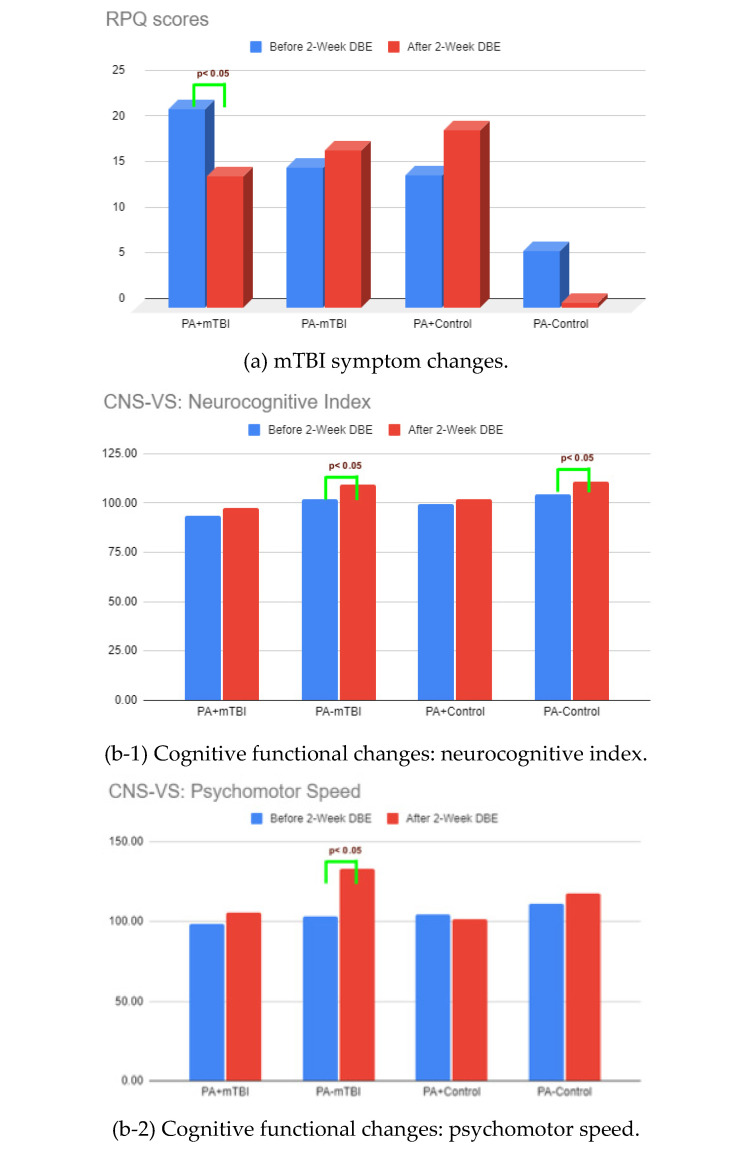

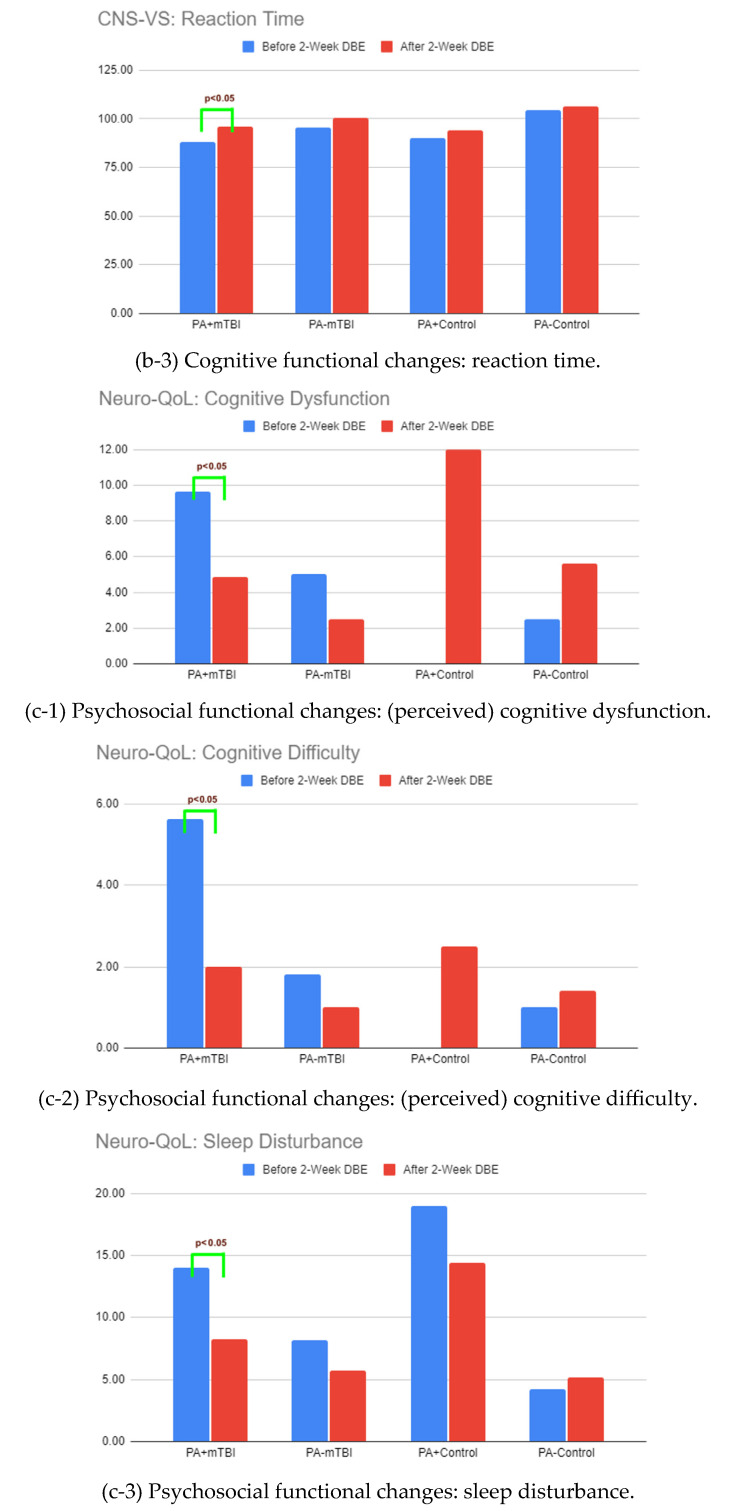

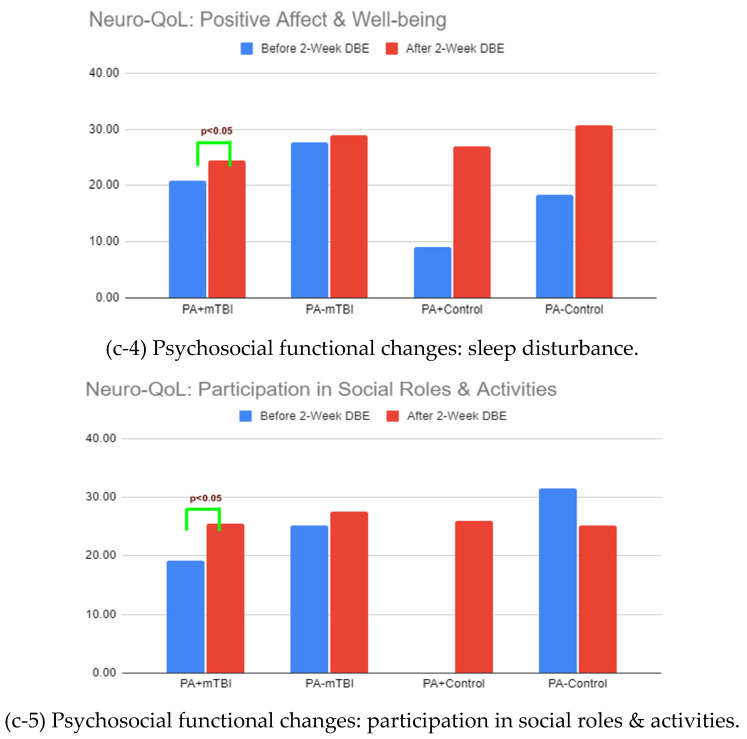
Functional changes in PA + mTBI, PA − mTBI, PA + control, and PA − control subgroups before and after the DBE.

**Figure 5:**
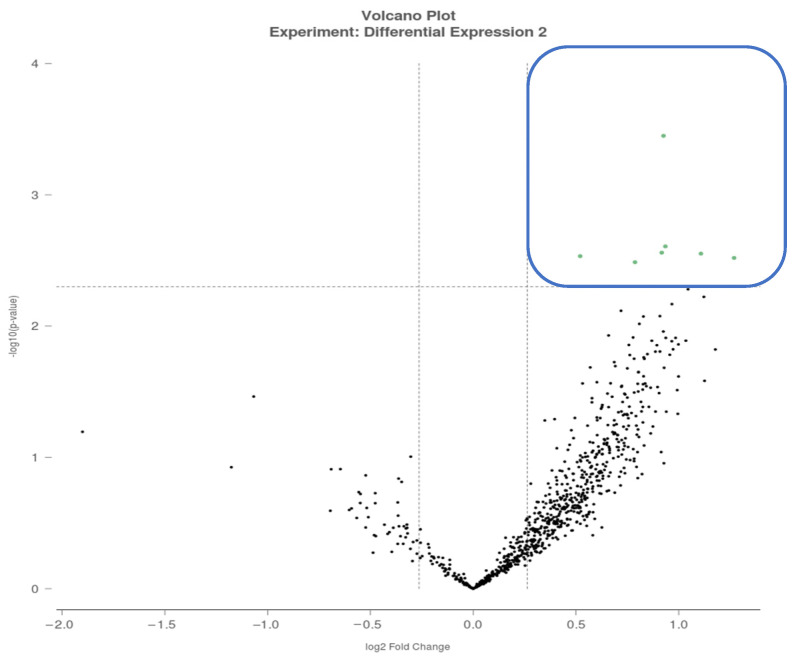
Volcano plot of differential expression between post- and pre-DBE in mTBI groups. Note: Reprinted from Rosalind Analysis, original Copyright © 2021 ROSALIND, Inc.

The PA + mTBI subgroup reported larger increases in scores across multiple domains following the DBE participation (Figure 4; Supplementary Table S3). These included reductions in the RPQ total symptom scores, improvements in the CNS-VS reaction time, and significant decreases in the Neuro-QoL cognitive dysfunction and cognitive difficulty (*p*’s < 0.01) (Figure 4(a,b-3,c-1,c-2)). Significant improvements were also observed in sleep disturbance, positive affect and well-being, and participation in social roles and activities (*p*’s < 0.05) (Figure 4(c-3–c-5)).

#### 4.4.2. PA − mTBI Subgroup

The PA − mTBI subgroup showed significant increases of scores in objective cognitive performance measures, including increases in the CNS-VS neurocognitive index, psychomotor speed, and processing speed (*p*’s < 0.05) (Figure 4(b-1,b-2)). No significant changes were observed in psychosocial symptom domains in this subgroup.

#### 4.4.3. Control Subgroup

Among the controls, the PA − control subgroup exhibited a significant improvement in CNS-VS Neurocognitive Index scores following the intervention (*p* < 0.05). No other significant within-group changes were observed in the cognitive, psychosocial, or symptom-based outcomes for either control subgroup.

### 4.5. Summary of Functional Recovery Patterns

Across the analyses, participants with both an mTBI and pre-injury psychosocial adversity (PA + mTBI) demonstrated the highest symptom burden at baseline but also exhibited the broadest pattern of improvement following DBE participation. These gains spanned cognitive efficiency, sleep, mood, and social functioning. In contrast, participants with an mTBI but without pre-injury adversity (PA − mTBI) showed more circumscribed improvements, primarily in objective cognitive speed and efficiency measures.

These findings indicate that pre-injury psychosocial adversity was associated with both heightened baseline impairment and differential patterns of post-intervention functional change.

### 4.6. Salivary microRNA Expression Changes Following the DBE

Exploratory analyses of salivary miRNA expression were conducted for participants who completed the 2-week DBE program. Differential expression analyses identified seven miRNAs that were differentially expressed following DBE participation only in the mTBI group (upregulated in post-DBE), using an unadjusted *p* < 0.005 threshold with 1.2+ fold change estimates (in the square of Figure 5). No miRNAs met the same criteria for differential expression among the control participants. This observation is associated with more functional changes in mTBI post-DBE participation compared with the controls, which was described in previous sections.

#### Differential miRNA Expressions in the mTBI Group

In the present study, miRNA findings are reported descriptively as exploratory signals intended to identify candidate markers for future validation. Among seven salivary miRNAs downregulated following the DBE participation in mTBI group (Table 2), the most robustly downregulated miRNA was *hsa-miR-7-5p*, with a 1.9-fold reduction from pre- to post-intervention (unadjusted *p* < 0.001). *hsa-miR-7-5p* is known for the negative regulation of amyloid-beta clearance, increasing amyloid-beta aggregation that is closely related with the cognitive decline in Alzheimer’s disease [32]. Additional significantly downregulated miRNAs included those implicated in neuronal development, synaptic regulation, and neurodegenerative disease pathways.

**Table 2:** Seven saliva miRNAs upregulated post-DBE in mTBI group.

miRNA	unadj. *p*	FC ^1^	Diseases Associated
*hsa-miR-7-5p*	0.000355	1.89975	Hepatocellular carcinoma, ovarian cancer, NSCLC, gastric cancer, negative regulation of amyloid-beta clearance, bipolar disorder, and negative ischemic stroke outcomes
*hsa-miR-454-3p*	0.003259	1.72569	Suppression of glioblastoma, lung cancer, bladder cancer, and pancreatic ductal adenocarcinoma
*hsa-miR-769-3p*	0.003024	2.41035	Inhibition of tumor progression in glioma
*hsa-miR-512-5p*	0.002759	1.88856	Head and neck squamous cell carcinoma
*hsa-miR-663a*	0.002806	2.15542	SLE, ovarian cancer, and breast cancer
*hsa-miR-582-5p*	0.00247	1.91205	Mesothelioma, leiomyoma, functioning pituitary adenocarcinoma, pituitary gland disease, and bladder disease
*hsa-miR-374b-5p*	0.002932	1.43436	Japanese encephalitis virus (upregulates this miRNA, resulting in inflammation)

Note: ^1^ FC—fold change.

Several of the identified miRNAs in the mTBI group post-DBE participation have been previously reported in association with neurodevelopmental and neurological processes in prior literature. Genes regulated by 6 to 7 of these seven downregulated miRNAs in mTBI include *SETD5*, *NFIB*, *FOXP1*, *ZNR148*, and *AP1G1*. Particularly, *SETD5* and *AP1G1*, which have been associated with neurodevelopmental regulation, synaptic function, and neurodegenerative mechanisms. Several of the identified miRNAs are associated with amyloid-beta processing, neuronal stress response, and cellular trafficking.

## 5. Discussion

This pilot study examined feasibility of the DBE program—a brief, home-based dual-task cognitive–walking exercise—along with patterns of cognitive, psychosocial, and exploratory biological changes observed following DBE participation among APIs with and without a history of self-reported mTBI, while accounting for pre-injury psychosocial adversity. The DBE intervention may represent a scalable and accessible approach to rehabilitation. Several descriptive observations emerged. First, participants with both mTBI and pre-injury adversity demonstrated the greatest baseline burden across cognitive, sleep, and psychosocial domains. Second, despite this vulnerability, following participation in the DBE, these subgroups (i.e., mTBI, PA + mTBI) exhibited broader patterns of pre–post change across the cognitive function, including reaction time and processing speed, psychosocial function, and overall post-injury symptom scores. The PA + mTBI subgroup showed the widest range of observed changes following DBE participation. Third, exploratory changes in salivary miRNAs were observed only among participants with mTBI and co-occurred with functional changes. Identified miRNAs following DBE participation in the mTBI group have been previously reported in association with neurocognitive development-related genes. Together, these findings suggest that pre-injury psychosocial adversity may be associated with baseline symptom burden and with descriptive patterns of changes following rehabilitation, while underscoring the feasibility of a brief, remotely delivered dual-task intervention in an understudied population.

### 5.1. Pre-Injury Psychosocial Adversity and Baseline Vulnerability

Consistent with the Stress–Diathesis Model, participants with pre-injury psychosocial adversity exhibited greater baseline cognitive and psychosocial symptoms, particularly when combined with a history of mTBI. Importantly, adversity was conceptualized in this study as a contextual modifier of symptom experience and recovery, rather than as a determinant of injury severity or neuropathology. The PA + mTBI subgroup exhibited greater impairments in sleep, anxiety, depression, cognitive difficulty, emotional dysregulation, fatigue, and social participation relative to other subgroups. Baseline differences were observed primarily in self-reported psychosocial domains, whereas objective cognitive performance did not differ significantly across subgroups prior to DBE participation. This dissociation highlights the importance of incorporating psychosocial context into mTBI assessment and care, as reliance on injury characteristics or performance-based measures alone may underestimate the symptom burden in trauma-exposed individuals.

It is important to acknowledge that pre-injury adversity was assessed retrospectively using self-report measures, which may be subject to recall bias. Additionally, adversity exposure was heterogeneous in type, timing, and severity between API participants in this study. As such, findings related to adversity should be interpreted as descriptive and hypothesis-generating rather than definitive evidence of causal influence.

### 5.2. Differential Patterns of Functional Changes Following Dual-Task Training

Following DBE participation, both the mTBI and control participants showed increases in objectively measured cognitive flexibility and executive function as assessed by CNS-VS Remote Testing. Additional increases in psychomotor and processing speed, as well as changes in perceived depression, sleep disturbance, and social participation, were observed descriptively within the mTBI group. These mTBI-specific patterns may be associated with the fact that dual-task training engages neural systems that were disrupted by mTBI. Prior literature has linked dual-task capacity to broader neurological functioning [19,21].

Participants with both mTBI and pre-injury adversity (PA + mTBI) demonstrated broader pre-post changes across multiple domains, including mTBI-related symptoms, reaction time, cognitive measures, sleep, positive affect, and social participation. Participants with mTBI but without pre-injury adversity (PA − mTBI) showed more circumscribed changes, primarily in objective cognitive performance indices, such as psychomotor speed and processing speed. Control participants exhibited minimal changes. These differential subgroup patterns may suggest that individuals with greater baseline burden may exhibit greater observable change following structured intervention; however, this interpretation must be approached cautiously.

This study employed a single-arm pre–post design without a non-intervention control group, which limits causal inference. Practice effects, expectancy effects, regression to the mean, and nonspecific effects of repeated testing cannot be ruled out. Although certain patterns were observed more prominently within mTBI subgroups than controls, the design does not allow definitive attribution of changes to the intervention. Accordingly, findings should be interpreted as preliminary patterns warranting confirmation in adequately randomized controlled trials.

### 5.3. Exploratory Salivary microRNA Findings

Exploratory analyses identified salivary miRNA changes following DBE participation exclusively among participants with an mTBI. Seven miRNAs, including *has-miR-7-5p*, were differentially expressed post-DBE with an unadjusted *p* < 0.005 with fold changes > 1.2. Prior literature has reported these miRNAs in association with neurodevelopmental and neurological processes. Five genes (*SETD5*, *NFIB*, *FOXP1*, *ZNR148*, and *AP1G1*) regulated by the identified miRNAs, including *hsa-miR-7-5p*, have been described in relation to neurocognitive development.

These findings are notable in that no similar changes were observed in the control participants; however, several limitations constrain interpretation. First, the miRNA analyses were exploratory, uncorrected for multiple comparisons, and conducted in a small sample. Additionally, salivary miRNAs represent peripheral signals and may not directly reflect central nervous system processes. Lastly, no independent molecular validation (e.g., qPCR) was performed.

Therefore, the miRNA findings should be viewed as hypothesis-generating indicators of potential biological processes that co-occurred with functional changes, rather than as evidence of mechanistic pathways or disease modification. The present results may contribute to identifying candidate miRNAs for future investigation.

### 5.4. Implications for Trauma-Informed Rehabilitation

This study focused on API participants, a population that remains underrepresented in mTBI research. Importantly, the inclusion of APIs addresses gaps in representation and contributes to the generalizability of rehabilitation research. It should be noted that the present study was not designed or powered to test differential biological responses or treatment effects by race or ethnicity.

APIs may experience unique barriers related to access, stigma, and underutilization of post-acute care, making the feasibility of home-based, remotely delivered interventions clinically relevant. The broader range of observed changes within PA + mTBI subgroup highlights the potential importance of trauma-informed approaches in mTBI rehabilitation. Rather than viewing pre-injury adversity as a static risk factor, these findings suggest that vulnerability may also confer heightened responsiveness to structured, goal-directed interventions. Accessible rehabilitation approaches with remote and home-based delivery formats may be acceptable and feasible in underserved populations by reducing barriers related to access, stigma, and logistical constraints.

### 5.5. Limitations and Future Directions

This study has several limitations. First, given the pilot nature of the study, the design was intended to evaluate feasibility and characterize observed patterns of change rather than to establish causal effects of the intervention. The single-arm pre–post design without a non-intervention control group limits causal inference, and nonspecific effects of repeated testing cannot be ruled out. Second, the small sample size and exploratory design limited the statistical power. Analyses were conducted to describe observed patterns rather than to test definitive hypotheses. Statistical significance was interpreted cautiously, and results are reported descriptively to inform future controlled studies rather than to support confirmatory conclusions. Third, the home-based format of the intervention introduced additional variability. Adherence and task performance were not directly supervised or controlled, and participation occurred in diverse environments, which may have influenced engagement and outcomes despite standardized instructions, exercise logs, and reminder messages. Additional limitations include technical difficulties encountered during remote cognitive assessments that required retesting in a small number of cases.

An additional consideration is the reliance on self-reported mTBI history without independent medical record verification or documentation of acute injury parameters (e.g., GCS score, neuroimaging findings). While this limits stratification by injury severity, the present recruitment strategy intentionally reflects the epidemiologic profile of mTBI in community settings. Most mTBI cases (GCS 13-15) do not often require hospital visits or specialty follow-up, particularly among young adults, and therefore, are not consistently captured in clinical registries. Emerging literature suggests that a subset of individuals with initially “normal-appearing” mTBI may nevertheless report subtle but persistent cognitive, sleep, or psychosocial concerns years after injury. By recruiting community-dwelling individuals with remote mTBI histories, the present study aimed to characterize functional patterns within this underrepresented population rather than within a narrowly defined acute clinical cohort. Accordingly, observed heterogeneity in time since injury and number of prior injuries reflects real-world variability typical of mild TBI populations outside hospital-based settings.

Nonetheless, the absence of restriction to a specific cause or type of acquired brain injury in this study may further contribute to heterogeneity in post-injury symptom manifestations and/or post-DBE outcomes. Finally, exploratory salivary microRNA analyses were conducted without correction for multiple comparisons and without independent molecular validation, limiting biological interpretation.

Collectively, these limitations highlight the need for larger, randomized, and methodologically rigorous investigations to validate and extend the present findings. Future research should build upon the preliminary findings from the present study with larger and more diverse samples. Incorporating in-person objective sensorimotor assessments, longer follow-up periods, and independent biomarker validation will be critical. Integration of mobile health platforms may enhance scalability, personalization, and adherence while reducing access barriers. Examining individuals closer to the time of injury, such as athletes within one-year post-mTBI, may further clarify intervention responsiveness across recovery stages.

## 6. Conclusions

This pilot study examined feasibility and exploratory patterns of functional and biological change observed following participation in a brief, remotely delivered home-based dual-task cognitive–walking program among API adults with and without a history of mTBI. Participation in the DBE was associated with descriptive increases in selected cognitive and psychosocial measures, particularly among individuals with both an mTBI and pre-injury psychosocial adversity.

Participants with greater baseline symptom burden demonstrated broader patterns of observed change following the intervention, highlighting the potential relevance of trauma-informed rehabilitation approaches. Exploratory changes in salivary miRNAs co-occurred with functional changes among participants with an mTBI, generating hypotheses for future investigation.

Taken together, these results support the feasibility and acceptability of a remotely delivered, home-based dual-task intervention in an understudied population and underscore the importance of incorporating psychosocial context and pre-injury adversity into mTBI rehabilitation research. Larger, randomized, and methodologically rigorous studies are needed to confirm these preliminary observations, validate biological markers, and evaluate the effectiveness of scalable, trauma-informed rehabilitation strategies for diverse populations affected by mTBIs.

## Figures and Tables

**Table 1:** Demographics, pre-injury adverse experience, and symptom score differences (*n* (%) or M ± SD) between mTBI and healthy control participants.

Characteristic	Overall (*N* = 21)	mTBI (*n* = 14)	Control (*n* = 7)	*x*^2^ or *t*	*p*
Age	22.86 ± 3.81	22.14 ± 3.48	24.29 ± 4.31	−1.230	0.234
Gender					
Females	14 (66.7)	9 (64.3)	5 (71.4)	0.000	1.000
Males	7 (33.3)	5 (35.7)	2 (28.6)		
Education					
High school graduate	9 (42.9)	7 (50.0)	2 (28.6)	1.625	0.654
Associate degree	4 (19.0)	2 (14.3)	2 (28.6)		
Bachelor’s degree	4 (19.0)	3 (21.4)	1 (14.3)		
Graduate degree	4 (19.0)	2 (14.3)	2 (28.6)		
Marital status					
Single	9 (42.9)	5 (35.7)	4 (57.1)	1.550	0.461
In a relationship	10 (47.6)	7 (50.0)	3 (42.9)		
Married	2 (9.5)	2 (14.3)	0 (0.0)		
Employment status					
Employed full-time	6 (28.6)	5 (35.7)	1 (14.3)	4.800	0.187
Employed part-time	2 (9.5)	0 (0.0)	2 (28.6)		
Unemployed	3 (14.3)	2 (14.3)	1 (14.3)		
Student	10 (47.6)	7 (50.0)	3 (42.9)		
Income					
Less than $19K	6 (28.6)	5 (35.7)	1 (14.3)	5.850	0.321
$20K–$29K	3 (14.3)	1 (7.1)	2 (28.6)		
$30K–$39K	2 (9.5)	2 (14.3)	0 (0.0)		
$50K–$74K	3 (14.3)	2 (14.3)	1 (14.3)		
$75K–$99K	5 (23.8)	2 (14.3)	3 (42.9)		
$100K–$149K	2 (9.5)	2 (14.3)	0 (0.0)		
THSs ^1^					
HMS	9.19 ± 11.43	8.64 ± 7.41	10.29 ± 17.73	−0.303	0.765
TS	1.90 ± 2.21	2.36 ± 2.41	1.00 ± 1.53	1.353	0.192
PPD	0.95 ± 1.88	1.43 ± 2.17	0.00 ± 0.00	2.459	**0** **.029**
mTBI-related symptoms					
RPQ	15.86 ± 11.19	19.50 ± 11.20	8.57 ± 7.25	2.332	**0** **.031**
NSI	19.52 ± 12.18	22.00 ± 12.02	14.57 ± 11.76	1.344	0.195

Notes: ^1^ THS—Trauma History Score; HMS—high magnitude stressor; TS—traumatic stressor; PPD—persistent post-traumatic event; RPQ—Rivermead Post Concussion Symptom Questionnaire; NSI—Neurobehavioral Symptom Inventory. Bold values indicate statistical significance (*p* < 0.05).

## Data Availability

De-identified data supporting the findings of this study are available from the corresponding author upon reasonable request. Data sharing is subject to institutional review board approval and applicable ethical guidelines.

## Supplementary Materials

The following supporting information can be downloaded at: https://www.mdpi.com/article/10.3390/clinpract16040065/s1: Table S1. Means, standard deviations, and one-way analyses of variance in cognitive and psychosocial functions among the 4 groups before the DBE; Table S2. Means, standard deviations, and one-Way analyses of variance in cognitive and psychosocial func-tions among the 4 groups after the Daily Brain Exercise; Table S3: Statistically significant changes after the Daily Brain Exercise.

## Abbreviations

The following abbreviations are used in this manuscript:

ADL	Activity of daily living
APIs	Asian and Pacific Islanders
APNA	American Psychiatric Nurses Association
CTE	Chronic traumatic encephalopathy
CNS-VS	CNS Vital Signs
DBE	Daily Brain Exercise
DSM	Diagnostic and Statistical Manual of Mental Disorders
HMS	High-magnitude stressors
miRNA	MicroRNA
mTBI	Mild traumatic brain injury
Neuro-QoL	Quality of Life in Neurological Disorders
NIH-SSM	National Institutes of Health Symptom Science Model
PA+, PA−	Adversity-positive or adversity-negative
PPD	Persistent post-traumatic distress
PTSD	Post-traumatic stress disorder
RNA	Ribonucleic acid
TBI	Traumatic brain injury
TS	Traumatic stressor
UNLV	University of Nevada, Las Vegas
