# Supplementary material for: Pre-Injury Adversity, Functional Recovery, and Salivary microRNA Changes After a Dual-Task Exercise in Asians and Pacific Islanders with Mild Traumatic Brain Injury: A Feasibility Study
Source: Clin Pract. 2026 Mar 25;16(4):65. doi: 10.3390/clinpract16040065 (PMC13114543; doi:10.3390/clinpract16040065)
Supplement: Supplementary file 1 [file clinpract-16-00065-s001.zip › clinpract-4104468-supplementary.pdf]

Supplementary Table S1. Means, Standard Deviations, and One-Way Analyses of Variance in Cognitive and Psychosocial Functions among the 4 Groups Before the DBE.

Characteristic	PA+ mTBI (*n* = 9)	PA- mTBI (*n* = 5)	PA+ Control (*n* = 2)	PA- Control (*n* = 5)	*F*(3,17)	*p*
mTBI-related Symptom Scores						
RPQ[1]	21.78±7.51	15.40±16.20	14.50±0.71	6.20±7.36	2.594	.086
NSI	23.44±10.61	19.40±15.21	24.50±19.10	10.60±6.88	1.396	.278
CNS-VS (Cognitive Function)						
Neurocognitive Index	93.50±9.87	102.00±6.63	99.50±7.78	104.40±8.59	1.904	.170
Composite Memory	100.22±12.26	108.60±17.01	96.00±29.70	111.20±7.36	1.012	.412
Verbal Memory	90.89±14.32	99.20±21.95	84.50±17.68	104.40±8.99	1.210	.336
Visual Memory	107.22±14.57	112.80±14.03	107.00±28.28	113.00±9.38	.267	.848
Psychomotor Speed	98.89±12.23	103.20±16.02	104.50±13.44	111.20±13.68	.854	.484
Reaction Time	88.00±13.13	95.60±10.36	90.00±8.49	104.40±14.93	1.859	.175
Complex Attention	98.25±16.26	104.60±5.77	110.00	97.20±11.30	.766	.530
Cognitive Flexibility	84.22±13.22	97.80±10.55	95.50±4.95	98.20±16.86	1.752	.194
Processing Speed	95.22±11.12	101.20±9.94	101.00±12.73	100.29±12.33	1.250	.323
Executive Function	83.67±12.64	98.20±10.18	94.50±3.54	98.60±16.29	2.142	.133
Simple Attention	103.63±13.44	105.40±5.27	110.00±2.83	92.20±30.48	.718	.556
Motor Speed	101.11±15.68	103.20±17.34	105.00±11.31	108.60±12.93	.264	.850
Neuro-QoL (Psychosocial Function)						
Cognitive (Dys)Function	9.63±3.11	5.00±3.74	.	2.50±3.54	5.075	**.025**
Cognitive Difficulty	5.63±2.13	1.80±2.05	.	1.00±1.41	7.371	**.008**
Anxiety	[a]**14.44±6.06**	7.20±5.09	4.00±4.24	[a]**5.40±2.07**	5.591	**.007**
Depression	7.78±6.42	3.80±3.35	4.00±1.41	1.00±1.41	4.469	**.022**
Sleep Disturbance	[a]**14.00±5.98**	8.20±6.18	[b]**19.00±4.24**	[ab]**4.20±3.49**	5.360	**.009**
Emotional & Behavioral Dyscontrol	7.89±4.01	6.80±4.15	6.00±2.83	3.00±1.00	2.214	.135
Stigma	2.75±3.99	1.20±2.68	.	.00	.660	.535
Fatigue	17.75±3.45	8.20±7.79	.	8.00±5.66	5.771	**.018**
Positive Affect & Well-being	20.89±7.32	[a]**27.60±2.88**	[a]**9.00±0.00**	18.40±11.99	2.942	.063
Participation in Social Roles, Activities	19.13±5.72	25.20±4.44	.	31.50±0.71	5.612	**.019**

Note. [1] RPQ = Rivermead Post Concussion Symptom Questionnaire; NSI = Neurobehavioral Symptom Inventory

[a-b] Groups with same letter represent significant differences as tested by one-way ANOVA with *post hoc* analysis with Bonferroni correction.

Supplementary Table S2. *Means, Standard Deviations, and One-Way Analyses of Variance in Cognitive and Psychosocial Functions among the 4 Groups After the Daily Brain Exercise.*

Characteristic	PA+ mTBI (*n* = 7)	PA- mTBI (*n* = 4)	PA+ Control (*n* = 2)	PA- Control (*n* = 5)	*F*(3,14)	*p*
mTBI-related Symptom Scores						
RPQ[1]	14.43±10.66	17.25±18.10	19.50±27.58	0.60±1.34	3.741	.138
NSI	17.67±15.93	18.25±14.38	26.00±24.04	11.40±10.11	.493	.693

 https://doi.org/10.3390/clinpract16040065

CNS-VS (Cognitive Function)						
Neurocognitive Index	97.29±10.77	109.50±5.69	102.00±7.07	110.80±9.42	2.629	.091
Composite Memory	88.43±19.76	113.50±5.51	96.00±29.70	112.80±7.76	3.298	.065
Verbal Memory	94.43±21.09	110.25±9.11	88.00±22.63	112.40±10.78	1.923	.172
Visual Memory	88.14±16.87	111.50±11.50	104.00±24.04	109.00±10.79	2.870	.074
Psychomotor Speed	[a]**106.00±14.20**	[a]**133.00±13.04**	101.50±14.85	117.80±16.30	3.521	**.043**
Reaction Time	96.14±16.23	100.25±15.28	94.00±1.41	106.60±12.90	.620	.613
Complex Attention	101.29±11.25	98.00±4.24	114.00±9.90	105.40±7.80	1.581	.238
Cognitive Flexibility	95.14±13.84	103.00±10.03	103.00	110.00±13.62	1.394	.286
Processing Speed	102.00±10.58	120.75±6.60	101.50±16.23	111.80±10.26	3.282	.053
Executive Function	96.00±13.65	104.25±9.91	102.50±2.12	110.80±13.54	1.415	.280
Simple Attention	106.29±6.05	96.25±9.39	104.00±11.31	103.00±9.00	1.294	.315
Motor Speed	105.86±16.25	127.00±23.59	100.00±8.49	115.60±18.09	1.520	.253
Neuro-QoL (Psychosocial Function)						
Cognitive (Dys)Function	4.86±3.63	[a]**2.50±3.31**	12.00±1.41	[a]**5.60±3.78**	3.354	**.050**
Cognitive Difficulty	2.00±1.67	1.00±1.15	2.50±3.54	1.40±1.67	.435	.732
Anxiety	11.71±5.96	8.75±6.34	12.50±16.26	6.40±5.86	3.743	.645
Depression	7.00±6.71	3.25±3.59	11.00±15.56	3.20±4.49	3.858	.688
Sleep Disturbance	8.26±5.28	5.75±5.19	14.50±14.85	5.20±4.32	3.750	.721
Emotional & Behavioral Dyscontrol	6.86±2.79	2.50±2.65	11.00±15.56	5.20±3.27	3.703	.314
Stigma	1.71±2.98	0.50±0.58	7.50±10.61	1.00±2.24	3.659	.697
Fatigue	10.57±6.53	8.25±6.85	19.00±18.38	6.23±2.79	1.869	.181
Positive Affect & Well-being	24.43±6.37	29.00±2.94	27.00±12.73	30.80±5.67	1.082	.389
Participation in Social Roles, Activities	25.57±4.54	27.50±3.00	26.00±8.49	25.20±6.76	.155	.925

Note. [1] RPQ = Rivermead Post Concussion Symptom Questionnaire; NSI = Neurobehavioral Symptom Inventory

[a-b] Groups with same letter represent significant differences as tested by one-way ANOVA with *post hoc* analysis with Bonferroni correction.

Supplementary Table S3. Statistically Significant Changes After the Daily Brain Exercise.

**Characteristic**	**PA+mTBI (*n* = 7)**			**PA-mTBI (*n* = 4)**			**PA-Control (*n* = 5)**		
	diff. [1]	*t*	*p*	*diff.*	*t*	*p*	*diff.*	*t*	*p*
RPQ	7.26	2.533	.044						
CNS-VS (Cognitive Function)									
Neurocognitive Index				-6.75	-3.282	.046	-6.40	-4.459	.011
Visual Memory	19.00	2.657	.038						
Psychomotor Speed				-24.75	-3.430	.042			
Reaction Time	-9.00	-2.737	.034						
Processing Speed				-19.50	-4.544	.020			
Neuro-QoL									
Cognitive (Dys)Function	6.00	2.598	.048						
Cognitive Difficulty	4.60	4.960	.008						
Sleep Disturbance	7.86	3.611	.011						
Positive Affect & Well-being	-6.14	-3.403	.014						
Participation in Social Roles & Activities	-8.83	-3.540	.017						

https://doi.org/10.3390/clinpract16040065

Note. [1] diff = difference. A positive difference is a reduction in the function, and a negative difference is an increase or improvement in the function between before and after the 2-week exercise.
